# Understanding the needs of employees with non-communicable diseases regarding physical activity-related workplace health promotion programs: a qualitative study

**DOI:** 10.3389/fpubh.2026.1919436

**Published:** 2026-09-03

**Authors:** Andrea Schaller, Lena Katharina Spanio, Martin Lange

**Affiliations:** 1 Institute of Sport Science, Faculty of Human Sciences, University of the Bundeswehr Munich, Neubiberg, Germany; 2 Department of Fitness and Health, IST University of Applied Sciences, Düsseldorf, Germany

**Keywords:** chronic disease, exercise, non-communicable diseases, workplace health programs, workplace health promotion

## Abstract

**Introduction:**

The prevalence of non-communicable diseases (NCD) is rising and increasingly affects people of working age. Thereby, NCD burden those affected and challenge society, welfare systems and companies in many different ways. Workplace Health Promotion (WHP) is considered promising to improve and maintain employee health and physical activity (PA)-related WHP programs constitute a main area of action. This study aimed to better understand the needs employees with NCD have with regard to PA related WHP programs.

**Methods:**

In an exploratory approach, qualitative partly structured interviews were conducted with 15 employees with NCD. The interviews were analyzed using structuring content analysis with concurrent deductive and inductive main categories, dimensions and expressions.

**Results:**

Based on the interviews, four main categories were identified: “subjective health concept”, “workplace-related health challenges and coping strategies”, “experiences with WHP” and “wishes and needs in terms of PA-related WHP”. “Subjective health concept” comprised the personal definition and the personal relevance of health. “Workplace-related health challenges and coping strategies” could be divided into physical and psychological demands and reported strategies were physical activity, nutrition, a supportive social environment and relaxing activities. The “experiences with WHP” comprised type of program, reasons for participation, access channels and subjective evaluation. The “wishes and needs in terms of PA-related WHP” referred to specific PA-content, structural aspects and psychosocial aspects.

**Discussion:**

The findings provide insights into the experiences and needs of employees with NCD regarding PA-related WHP and provides recommendations for the further development of WHP tailored to specific target groups. Thereby, WHP for employees with NCD must not only be consistently adapted to the target group's work-related environment, but also take into account their mental, physical, and social limitations.

## 1 Introduction

Non-communicable diseases (NCD) not only burden those affected, but also challenge society and its welfare systems in many different ways (1, 2). In this regard, companies are exposed to considerable financial and social cost as well, e.g. due to continued payment of salaries and additional workload for colleagues during sick leave, productivity losses, reintegration measures, cost for recruiting and training replacement staff (3–5).

The global prevalence of NCD is rising and around 6 out of 10 young and 8 out of 10 midlife adults in the United States report one or more chronic conditions (6), while in Germany, around 50% of adults in working age suffer from at least one NCD (7, 8). The prevalence of NCD among the working population in the European Union is estimated around 25% (9–12). Diagnoses comprise cardiovascular diseases, cancer, chronic respiratory diseases, mental illness and, most frequently mentioned, musculoskeletal disorders (8). The challenge of dealing with chronic health conditions in the workplace is particularly relevant because young adults are also increasingly affected by NCD (6). Because of the impact of NCD on work capacity questions on how to maintain or improve individual work ability over the lifespan are of utmost importance for social insurers and companies.

Workplace initiatives and programs aiming at improving and/or maintaining employee health are considered promising (13, 14). In Germany, a systematic occupational health management (OHM) encompasses behavioral and organizational offers and focuses on the company's structures, processes and health-related outcomes (15). Thereby, OHM is based on four interrelated legal domains with varying degrees of obligation for employers and employees (15). The first domain, occupational safety, is a legal obligation for employers and employees (16, 17). The second domain, the German company-based return-to-work management, supports employees in the process of returning to work after a long period of sick leave and is mandatory for employers but voluntary for employees (18, 19). The third domain focusses on medical prevention measures for specific indications (e.g., musculoskeletal indications) that can lead to longer or more frequent sick leaves (20, 21). The fourth domain of OHM, workplace health promotion (WHP), focuses on promoting health-oriented behavior among employees, offering and participating WHP is voluntary for both groups—employers and employees and financed by statutory health insurance funds under certain conditions (15, 22). In this regard, the expenditure of statutory health insurance funds has increased four-fold over the last decade [2014: €68 million (23); 2024: €282 million (24)]. In this context, physical activity-related WHP programs constitute the main area of action for both behavior-oriented and environmental measures (24). Given the evidence regarding the positive health effects of physical activity (PA) this is not surprising: Physical inactivity is acknowledged as one of the five major risk factors of NCD (25–27) and the positive health effects of PA have been comprehensively proven in both prevention and therapy (28, 29). However, there is a paradox in that people with health impairments generally engage in less PA than healthy adults and therefore benefit less from the associated positive health effects (30). This is attributed to worse access and fewer programs for specific groups (31, 32). Considering the target groups of current WHP programs, it becomes apparent that WHP programs are rarely targeted at groups with health-related issues (5% of programs) or people with disability (3% of programs) (24) and also the evidence on WHP for employees with NCD in Europe is very limited (33).

Against the background of the increasing prevalence of NCD among employees as well as the potential of WHP and PA for this target group, the present study aimed to better understand the needs of employees with NCD regarding WHP programs. The related research question was: What needs do employees with NCD have with regard to PA related WHP programs?

## 2 Methods

### 2.1 Study design

In an exploratory approach, qualitative partly structured interviews were conducted at one time point in accordance with the COnsolidated criteria for REporting Qualitative research (COREQ) (34).

### 2.2 Participants and recruitment

Participant recruitment and data collection were conducted by one of the authors (LKS). Participants were recruited via posters and personal contact in the author's professional environment (health center). The selection sample comprised members and guests of the club who met the following inclusion criteria: (a) at least one NCD for at least 6 months, (b) current employment, (c) and sufficient language skills to participate in the interview. Fifteen persons (8 women and 7 men) with a mean age of 50 (±14) years (range: 27–65 years) participated in an interview. Interview durations ranged from 26 to 64 min [mean = 39 (±11) min]. The sample composition is presented in Table 1.

**Table 1:** Sample description and interview-length per group.

Interviewees (*n* = 15)	Value
Age (years)	
Mean (±SD)	39 (±14)
Minimum–maximum	27–65
**Gender:** female (*n*; %)	8 (53%)
NCD (Multiple answers allowed)	
• Cardiovascular disease (*n*; %)	4 (27%)
•Musculoskeletal disease (*n*; %)	12 (80%)
•Mental illness (*n*; %)	3 (20%)
•Other (*n*; %)	3 (20%)
**Employment:** Full-time (*n*; %)	9 (60%)
Highest level of education	
•No school-leaving certificate	0
•Moderate qualification (graduation after 8–10 school years)	1 (1%)
•15.6-15.2,-13.8242ptHigh qualification (graduation after at least 12 school years)	14 (99%)
Occupational field	
•Production	2 (13%)
•Technical	1 (7%)
•Transportation and Logistics	1 (7%)
•Business Services	1 (7%)
•Administration	5 (33%)
•Health, Social Services, and Education	5 (33%)
**Interview duration (minutes)**: Mean (±SD)	39 (±11)
Minimum–maximum	26–64

*n*, number; SD, standard deviation; NCD, non-communicable disease.

### 2.3 Interview guide

A problem-centered interview guide was developed following the SPSS-Principle [Collection (Sammlung) review (Prüfung), sorting (Sortierung), summarization (Subsumierung)] (35) and covered three main topics: (a) health challenges in everyday working life, (b) experiences with WHP programs, (c) wishes and needs for an WHP program. Open questions on these topics were collected and jointly formulated by the research team (LKS, AS). Comprehensibility and estimated interview duration were pilot-tested with researchers who did not have detailed knowledge about the study and the interview guide was then revised. The interview guide was developed in German and translated using established guidelines (36, 37) in English for publication purposes only. Topics and key questions are presented in Table 2.

**Table 2:** Topics and key questions of the interview guide.

Topic	Key questions
Health challenges in everyday working life	•What is a typical workday like for you? •What health challenges do you face during a typical workday?
Experiences with workplace health promotion programs	•What workplace health promotion programs offered by your company are you aware of? •What impact did these programs have on your work and your health?
Wishes and needs in terms of physical activity-related WHP	•Imagine you could design an exercise program to promote health in the workplace—what would it look like? •In view of your personal health condition, what would be important to you in a workplace health promotion program?

### 2.4 Data collection

The interviews were conducted from April to June 2025. All participants signed a written consent form before participating, in which they received written information about the objectives, content, and data protection provisions of the study. Participation was voluntary, and participants could withdraw at any time without giving reasons and without any disadvantages. The interviewer (LKS) had a bachelor's degree in health science was trained in qualitative research by AS and collected the data as part of her master's thesis. All interviews were conducted in German and the interviewees were informed that they could take a break whenever they wished. All interviews were conducted in a quiet room of the health center (see above). The interviews were digitally recorded and the respondents subsequently completed a short questionnaire with sociodemographic information. After 15 interviews (single interviews, no repeat interviews), no new information seemed to arise and the researchers decided to stop data collection due to the principle of data saturation.

### 2.5 Data analysis

The audio recordings were transcribed using the “noScribe” software and then manually revised to ensure accuracy of content. The transcription was carried out according to the content-focused transcription rules of Kuckartz (38). The interviews were analyzed using content-structuring qualitative content by Mayring (39). The analysis was conducted using a deductive-inductive approach: First, main categories were developed deductively on the basis of the interview guide and theoretical assumptions, then the category system was expanded and differentiated through inductive deductions from the material. The coding process was carried out manually by LKS on the basis of the transcripts in several rounds. Text passages were marked, coded, and assigned to a category system, which was documented in Excel and continuously revised. The coding system then was discussed with other researchers (AS, ML).

## 3 Results

**Figure 1:**
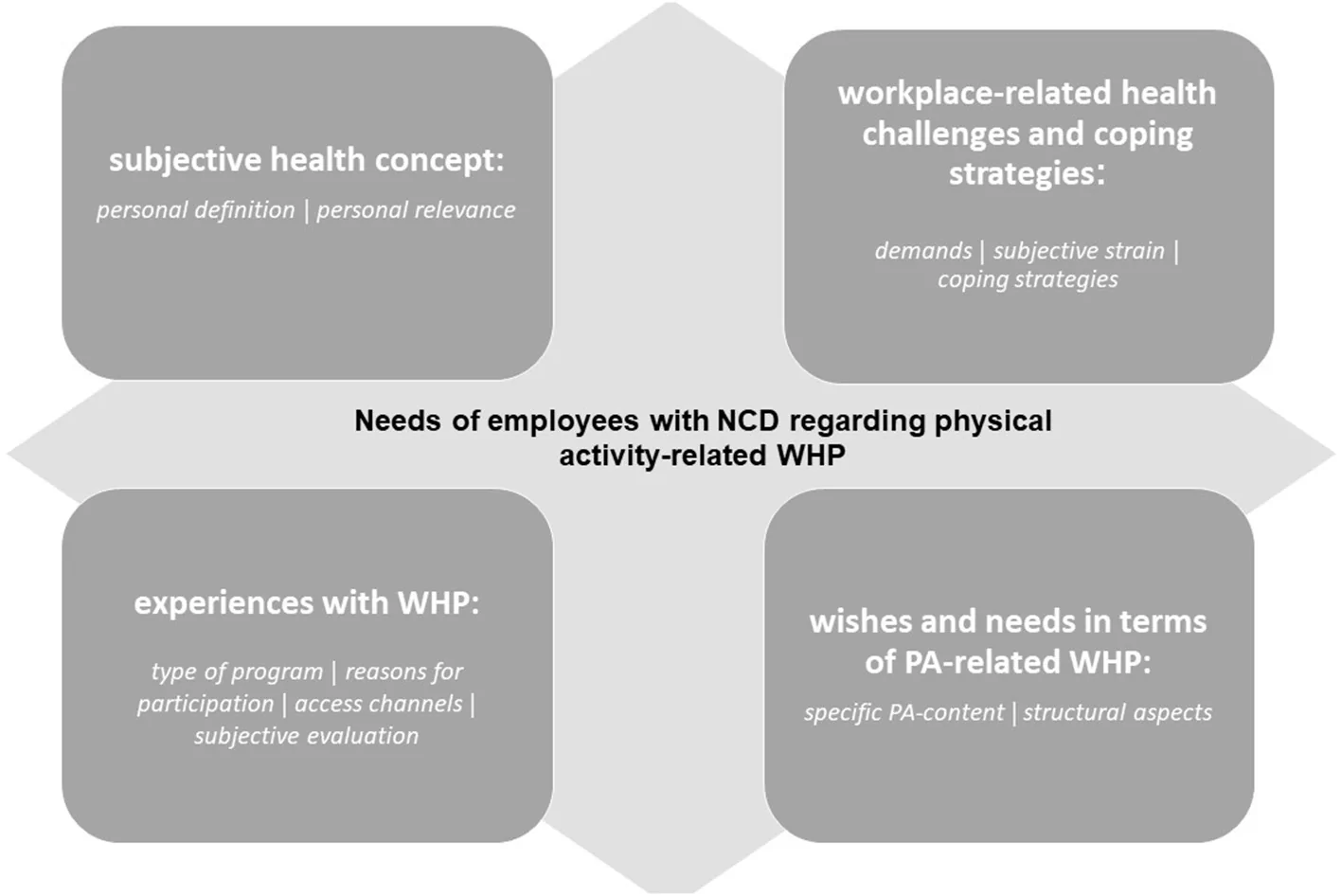
Main categories and dimensions.

The deductive-inductive content analysis showed four main categories: “subjective health concept”, “workplace-related health challenges and coping strategies”, “experiences with WHP” and “wishes and needs in terms of PA-related WHP” (Figure 1). Subsequently, the results are presented in relation to the respective main categories and dimensions.

### 3.1 Subjective health concept

The main category “subjective health concept” describes the individual perception of what constitutes health and how it influences life. The main category comprised two dimensions: *personal definition* and *personal relevance*.

The *personal definition* of health varied considerably. While some of the interviewees described health as the absence of illness, pain or symptoms (“*To live my life without symptoms, pain or impairments [...]” [I05]; “I would feel healthy, completely healthy, if I had no pain now [...]” [I13])*, others described a rather resource- and participation-oriented subjective concept of health that goes beyond the approach of absence of illness (“*If I am allowed to do what I want, if I am allowed to be myself [...]” [I10]; “So, in social interaction with people I like [...]” [I04]*).

*Personal relevance* referred to the importance of one's own health in everyday private and professional life. Thereby, the interview partners did not make a clear distinction between private and professional relevance (“*[...] It actually affects my leisure time much more, because I can't walk very far, I can't stand for long periods of time and there's not much I can do that I'd like to do [...]” [I07]*). The high relevance of one's own state of health in a professional context was described not only on a physical level, but also on a social:

“*So when I notice that I'm not feeling well, especially with back pain or neck pain. That's when you get annoyed at work, you get frustrated at work, you're not focused. [...] At some point, especially when you have a blockage like that, something can quickly slip out [...] that you wouldn't normally do when you're feeling well. And it's the same at home [...].” [I08]*

“*Of course, it's also important for others that you're healthy, because then you can contribute on a completely different energy level, whether it's in your family or simply in your everyday working life [...].” [I12]*

### 3.2 Workplace-related health challenges and coping strategies

This main category described the individual health challenges as a result of occupational strain and the strategies applied to cope with them. The subcategories identified were *demands, subjective strain* and *coping strategies*.

*Demands* could be divided into physical and psychological demands. In addition to forced postures, such as prolonged sitting (“*meaning essentially at the table. Bound, more or less [...]” [I09]*), physically demanding tasks and specific demands on the eyes due to screen work were also mentioned. In terms of psychological demands, work intensification, time pressure, high communication volume, constant availability, high attention and concentration demands as well as emotional demands, especially due to the atmosphere in the workplace and with supervisors, were reported.

“*and I have a lot of phone calls [...] where everything has to work quickly. [...] The department has to run, should run, no matter what.” [I08]*

“*So it's not just a case of working from nine to twelve and then going home. [...] At the weekend, [...] I have to be more or less on call [...].” [I13]*

“*So, being the center of attention […]. You have to constantly act and react. [...] conveying information, acting and reacting to disruptions or other things, that's really tough [...].” [I03]*

According to the interview partners, these physical and psychological demands were corresponding to *subjective strains*, e.g., pain and tension, physical exhaustion, emotional exhaustion, loss of motivation and restlessness.

“*[...] especially after eight hours on the job, you often lack the motivation to do anything else [...].” [I08]*

“*[...] that also affects motivation [...]. It just decreases [...].” [I07]*

“*[...] that you take things home with you. You think about them longer, or even when you're doing something completely different, like on the weekend or something, you think about them [...].” [I09]*

“*[...] and in the evening I notice that I have a headache, that my neck hurts [...]. And because of all the running around, now acute, this bursitis.” [I01]*

*Coping strategies* to compensate the subjective work-related strains were physical activity (“*[...] Getting up sometimes, doing something different“ [I01]; ”What helps me is balance, exercise, going outside, just going for a walk.” [I09])*, nutrition *(“No alcohol, little sugar, lots of protein, a certain amount of time between meals, time to digest [...]” [I11])*, a supportive social environment as well as relaxation and regeneration activities:

“*Well, handicrafts, for example [...], that calms me down quite nicely [...].” [I04]*

“*[…] music actually quite a lot, where you can then, yes, think about other things […].” [I09]*

“*[...] lying down for a moment, closing my eyes for a bit [...].” [I14]*

“*[...] meeting friends to balance things out, talking about other topics, not about work, but really about something that completely distracts you.” [I09]*

“*Yes, my colleagues are very supportive.” [I06]*

“*It really helps when you can talk about things [...].” [I07]*

### 3.3 Experiences with WHP

Experiences with WHP summarizes the interviewees' current and past experiences with WHP programs. The analysis identified the following dimensions: *type of program, reasons for participation, access channels* and *subjective evaluation* of the programs participated in.

Regarding the *types of programs* offered, respondents mentioned specific offers provided by external partners as part of their company's WHP, which employes could use for free or for a small fee *(“Yes, what's available are, of course, these sport groups or discounts for various clubs or fitness facilities […]” [I01]; “[…] the Jobrad actually counts as part of it too, because it gives you a chance to get some exercise in everyday life” [I10])*. Other types of programs mentioned included company sports *(“I go, again, with a group to play curling or bowling.” [I05])* and in-house sports programs with organized classes *(“Well, I knew they offered this kind of active lunch break. It's kind of a mix of gymnastics and a yoga class [...]” [I13])* as well as the use of company sports facilities, such as fitness rooms or gyms *(“there are also a lot of sports facilities there, so there's a swimming pool, there's a fitness center, there are gyms […]” [I07])*. In addition, one-time WHP initiatives such as challenges were also reported *(“So we tried something with a step challenge, [...] where groups sort of formed [...] and then counted their steps over a period of time—a few weeks. And in the end, the team with the most steps won [...]” [I09])*.

*Reasons for participation* were personal health and financial considerations as well as the proximity to the workplace and the quality respectively the variety of the WHP programs:

“*Because I enjoyed it and because it's definitely a financial benefit, too […].” [I05]*

“*I can exercise and get it counted toward my work hours […].” [I11]*

“*[…] I used this gym […] because it's right next door to the building, so I really just have to walk over there once […].” [I07]*

“*[…] I think it's really great because it's just so versatile, and yeah, you have so many options to choose from and the chance to try something different every now and then […].” [I14]*

Regarding the *access channels*, meaning how employees learned to know about WHP programs, especially an unclear procedure and the timing of the programs was named as reasons for not participating in WHP:

“*But every time I signed up, it never actually happened. Or it was already full […] and because of that, I never found out about it or found out too late […].” [I06]*

“*[…] because the timing was completely impractical with my part-time job, […] especially since it basically cut into my lunch break.” [I13]*

“*But there's deliberately very little promotion of these programs, which is why I'd say that none of us even knows what else is available […].” [I01]*

“*A colleague mentioned once, […] that a course was taking place again. But by then, of course, it was already too late […].” [I06]*

In contrast, also systematic dissemination of WHP offers were reported, e.g. by newsletters, intranet platforms *(“That went out on our intranet. Everyone saw it […]” [I02])* or placement at the workplace *(“we have a screen like that, and there, the programs are displayed […]” [03]; “HR provides information via newsletters” [I12])*.

The *subjective evaluation* of the WHP programs participated in seemed to depend on their impact on individual wellbeing and performance, comprising physical and mental effects:

“*So, in that sense, yes, whenever I've actively participated in the programs, I've always noticed that I feel better. Both emotionally and physically […].” [I01]*

“*[…] for my back—that's my weak spot—it's just better.” [I06]*

“*So, as I said, the exercise was very, very, very important. It has really given me a lot now.” [I10]*

“*Well, as I said, without the fitness department, I'm 100% sure I wouldn't be able to do the work the way it's done now, nor would I want to [...].” [I08]*

“*So definitely for the training effect, because training together actually boosts performance if you want to keep up—since you're training together […].” [I11]*

However, not all participants report positive effects *(“Actually, I haven't noticed anything yet […]” [I07]; “[…] what actually helps me with the pain […], in that regard, I don't really feel supported by my employer […]” [I13])* and also the sustainability of the intervention regarding the transfer to everyday life was questioned *(“So this feeling—what you've been told, what you've learned, what you've experienced positively—you carry that with you for a while, and then it gets lost again in everyday life” [I01])*. Beyond, especially the quality of the WHP offer (“*But then I stopped because I figured no one was watching what I was doing, and that's important to me [...]” [I07]*) and psychosocial aspects showed negative rating (“*That's what stopped me—I'm relatively high up in the hierarchy. And I didn't really want to be jumping around in the video with the others […]” [I02]*).

### 3.4 Wishes and needs in terms of PA-related WHP

The specific requests regarding PA-related WHP included *specific PA-content, structural aspects*. The preferred content of s*pecific PA offers* encompassed both the physical and the psychosocial aspects:

“*Definitely with back exercises, […] it really starts from the top of your head down to your little toe—moving your whole body and, yeah, working through everything […].” [106]*

“*A class like, for example, […] Zumba or yoga—I'd like something like that, but also the option to just do something on my own there without having to stick to set times […].” [I04]*

“*[…] a ping-pong table would be nice, too. Maybe a football table for coworkers […] something you can do together […].” [I04]*

“*creating a space where you can connect with others, especially around exercise […].” [I10]*

“*I think mental health is actually a very important part of it, because if you have mental health issues, you can't always exercise […].” [I10]*

Regarding *structural aspects*, the importance of group activities was pointed out, but also the desire for individual sessions with therapeutic expertise due to their specific health impairments was reported:

“*I have to say, it really does make a difference—it's not just about going to therapy or treatment all the time; there's also a bit of a fun factor to it, and that's incredibly important.” [I13]*

“*Well, in our case, I'd see that as a group activity. We always work as a team, so we can support each other and learn new things […].” [I11]*

“*[…] I also need a massage or something like that, because I sometimes have pain in places I can't reach myself, and I can't relieve those muscle spasms with exercises either—I need a physical therapist for that […].” [I13]*

“*[…] so what would probably be good for me personally […] is a one-on-one session.” [I01]*

## 4 Discussion

The findings of the present study provide insight into the workplace related health challenges that employees with NCD face and how they perceive the WHP programs offered by their respective companies. Four main categories were identified: “subjective health concept”, “workplace-related health challenges and coping strategies”, “experiences with WHP” and “wishes and needs in terms of PA-related WHP”. “Subjective health concept” comprised the personal definition and the personal relevance of health. While the personal definition of health varied considerably, the personal relevance was consistently described as high for both work and leisure time. “Workplace-related health challenges” could be divided into physical and psychological demands and reported “coping strategies” were physical activity, nutrition, a supportive social environment and relaxing activities. The “experiences with WHP” were diverse regarding the type of program, the reasons for participation, the access channels and as well as subjective evaluation of the programs. The “wishes and needs in terms of PA-related WHP” referred to specific PA-content, structural aspects and psychosocial aspects.

The workplace is described as a key point of access with potential to reach vulnerable groups and thus contribute to health equity (14, 40). Since reducing socially determined and gender-based inequalities in health opportunities is a central objective of the services funded by health insurance to promote self-directed, health-oriented behavior among individuals (health promotion) and to prevent and reduce health risks (primary prevention) (22), this aspect is of particular relevance. Especially, since people with NCD tend to have lower health literacy on average (41), the group of people with NCD should also be given greater attention and explicitly addressed in WHP. Despite their underlying conditions, people with NCD also retain physical and mental health potential that can be developed and must be promoted to maintaining long-term work capacity (42). Just as the 2015 Act on Strengthening Health Promotion and Prevention determines that preventive care services must also be provided in inpatient and outpatient care facilities to address the needs of persons requiring care (43, 44), the potential of WHP to reach and address the needs of employees with NCD needs to be better exploited.

In this regard, the conceptual approach of WHP also offers promising possibilities, also for employees with NCD (45, 46). The Luxembourg Declaration describes WHP as a corporate strategy aiming at preventing physical and mental illness in the workplace, strengthening health potential, and improving wellbeing in the workplace (47). WHP interventions range from behavioral measures with active participation (e.g., courses or seminars) to organizational measures such as consultations, analyses, inspections, and establishing organizational structures such as a health committee (45). Our results showed, that health was described as fundamental to wellbeing, quality of life and participation. Regarding the personal relevance of health, the participants did not show a clear distinction between private and professional life and the personal definition of health varied considerably. However, implementing WHP for employees with NCD requires that programs also take into account potential condition-related limitations and risk factors, as well as specific psychosocial aspects and self-management of chronic disease (42).

Our results showed, that employees with NCD have different and/or additional needs regarding WHP compared to the general employee population (“*[…] I also need a massage or something like that, because I sometimes have pain in places I can't reach myself, and I can't relieve those muscle spasms with exercises either—I need a physical therapist for that […]” [I13]; “[…] so what would probably be good for me personally […] is a one-on-one session” [I01])*. However, the focus so far has been primarily on the integration of health management, work organization, and long-term employability. However, not all requested services (e.g., massage, one-on-one services) meet the quality criteria specified in the Prevention Guidelines (15). Beyond, our results showed, that respondents have had varied experiences with access to WHP and the respective programs. On the one hand, there were respondents who have already accessed and benefited from such programs. On the other hand, some respondents did not benefit *(“Actually, I haven't noticed anything yet […]” [I07])* or reported a lack of transparency or a complete absence of such initiatives. This even gave the impression that the employer is deliberately withholding information (“*But there's deliberately very little promotion of these programs, which is why I'd say that none of us even knows what else is available […]” [I01])*. Besides the need of a strategic and transparent communication of WHP initiatives (48), our results refer both to the need for services that are sensitive to impairments and tailored to specific target groups, as well as to the accessibility of these services.

Health insurers and employers should consider whether and to what extent the specific needs of persons with NCD can be addressed and supported in the context of WHP. On the one hand, this could help to keep employees with NCD in the workforce longer. On the other hand, it could also make a long-overdue contribution to bridging the “silo mentality” in Germany's sector-based healthcare system and the outdated, rigid distinction between primary, secondary, and tertiary prevention services. A greater focus on the multidimensional approach of physical activity programs (49), with greater consideration of psychosocial aspects, could also make a substantial contribution to this objective. This, in turn, would meet the quality requirements of the Prevention Guidelines, which base physical activity programs on the health-oriented sports model (15). This model formulates six core objectives: strengthening physical health resources, strengthening psychosocial resources, reducing risk factors, managing health complaints, and fostering a commitment to sports and physical activity an improving movement conditions (50). The authors suggest that the objectives of strengthening psychosocial resources, managing health complaints, and fostering engagement with sports and physical activity are often insufficiently addressed in WHP programs.

A strength of our study lies in the exploration of the needs and experiences of a target group that has received little attention to date. At the same time, some limitations need to be taken into account. One limitation of this study is that, due to its qualitative design and small sample size, it cannot provide generalizable results or conclusions. Hence, the number of interviews is within established thresholds of sample size recommendations (51, 52). Secondly, due to recognizability in a small, monocenter qualitative study, we could not present detailed socio-demographic information. Thirdly, the participant recruitment is a limitation, as participants were recruited via personal contact and only in one health center. Fourth, it is important to note that the sample is composed of individuals with NCD who have prior experience with physical activity, as the participants were recruited through a health and fitness club and therefore already have extensive experience with physical activity. The results are therefore not transferable to physically inactive employees with NCD and their experiences and needs.

## 5 Conclusion

WHP programs for employees with NCD must not only be consistently adapted to the target group's work-related environment but also take into account their mental, physical, and social limitations in order to ensure their opportunities for participation in the workforce and to enhance the long-term effectiveness of interventions. Since reducing inequalities in health opportunities is a central objective of the services funded by health insurance and employees with NCD make up a large proportion of the working population, this vulnerable target group must be given more attention within the scope of WHP.

## Data Availability

The raw data supporting the conclusions of this article will be made available by the authors, without undue reservation.
